# Development and Optimization of an Eplerenone-Loaded Liposomal In Situ Gel for Enhanced Intranasal Delivery

**DOI:** 10.3390/pharmaceutics18060678

**Published:** 2026-05-29

**Authors:** Juste Baranauskaite, Ipek Ceken, Asta Kubiliene, Rima Jurate Gerbutaviciene, Ebru Türköz Acar, Cetin Tas

**Affiliations:** 1 Department of Pharmaceutical Technology, Faculty of Pharmacy, Yeditepe University, 34755 Istanbul, Turkey; juste.ortasoz@yeditepe.edu.tr (J.B.); ipek.ceken@std.yeditepe.edu.tr (I.C.); cetin.tas@yeditepe.edu.tr (C.T.); 2 Department of Analytical and Toxicological Chemistry, Faculty of Pharmacy, Academy of Medicine, Lithuanian University of Health Sciences, 50162 Kaunas, Lithuania; 3 Department of Drug Technology and Social Pharmacy, Faculty of Pharmacy, Academy of Medicine, Lithuanian University of Health Sciences, 50162 Kaunas, Lithuania; rima.jurate.gerbutaviciene@lsmu.lt; 4 Department of Pharmaceutical Analytical Chemistry, Faculty of Pharmacy, Yeditepe University, 34755 Istanbul, Turkey; ebru.acar@yeditepe.edu.tr

**Keywords:** Eplerenone, liposomes, in situ gel, intranasal delivery, encapsulation efficiency, computational analysis

## Abstract

**Objectives**: this study aimed to develop and optimize an intranasal delivery system for Eplerenone (EPL) by incorporating Eplerenone-loaded liposomes (Elip) into an in situ gel system (Elip-GG). The goal was to prolong the residence time of the drug in the nasal cavity and ensure sustained release. **Methods**: Elip and unloaded liposomes were prepared using the thin-film hydration method. Key formulation variables such as encapsulation efficiency (EE%), mean particle size (MPS), polydispersity index (PDI), and zeta potential (ZP) were optimized. The Elip was then incorporated into a gellan gum (GG) in situ gel to form Elip-GG. The Elip-GG formulation was evaluated based on parameters such as pH, viscosity, rheological behavior, mechanical properties, and in vitro release. **Results**: the optimal Elip formulation exhibited an EE of 86.3%, a mean particle size of 86.56 nm, a PDI of 0.29, and a ZP of −29.86 mV. The cumulative drug release from the Elip-GG formulation exceeded 93% after 2.5 h. The Elip-GG formulation significantly increased the sustained release of Eplerenone when administered intranasally, offering a promising alternative to oral and parenteral delivery methods for hydrophilic antihypertensive drugs.

## 1. Introduction

Nanoscale drug delivery systems have emerged as a cutting-edge strategy to overcome the limitations of conventional drug formulations, particularly the poor bioavailability of many active pharmaceutical ingredients [1]. Among these, vesicular carriers such as liposomes provide significant advantages, including protection of encapsulated drugs from chemical and enzymatic degradation, improved stability, and enhanced permeability across biological membranes [2]. These systems are especially beneficial for drugs with suboptimal physicochemical properties, enabling improved therapeutic performance.

Hypertension and chronic heart failure remain major global health challenges, requiring effective long-term pharmacotherapy. Eplerenone (EPL), a selective mineralocorticoid receptor antagonist, is widely used in the management of these conditions due to its cardioprotective effects and reduced risk of adverse endocrine side effects compared to earlier agents. However, EPL is classified as a Biopharmaceutical Classification System (BCS) Class II drug, characterized by low aqueous solubility and high permeability, which significantly limits its oral bioavailability and therapeutic efficiency. Therefore, alternative delivery strategies are required to enhance its clinical performance.

The intranasal route has gained considerable attention as a non-invasive and efficient pathway for systemic drug delivery. The nasal cavity offers several physiological advantages, including a large surface area, highly vascularized epithelium, and a porous endothelial membrane, allowing rapid drug absorption and avoidance of first-pass metabolism [3]. Intranasal administration enables a faster onset of pharmacological action, reduced dosing requirements, and minimized systemic side effects [3,4]. Additionally, the incorporation of mucoadhesive polymers into nasal formulations can further enhance drug absorption by prolonging residence time at the site of administration [5,6]. Despite these advantages, rapid mucociliary clearance remains a key limitation, necessitating formulation strategies that improve retention and drug permeation.

To address these challenges, advanced drug delivery systems combining nanoscale carriers with mucoadhesive platforms have been explored. Liposomal systems can penetrate the mucus barrier and facilitating drug transport across the nasal epithelium, while in situ gelling systems undergo sol–gel transition upon exposure to physiological conditions, thereby increasing formulation residence time [7,8,9]. Also, it is very important to choose the right gelling agent while preparing the in situ gels. Gellan gum was selected as the gelling agent due to its ion-sensitive in situ gelation, biocompatibility, and proven suitability for intranasal drug delivery systems. In the presence of physiological ions such as Na^+^ and Ca^2+^ in nasal fluids, gellan gum undergoes a rapid sol–gel transition, allowing administration as a low-viscosity solution that subsequently forms a gel in situ. This transition enhances nasal residence time, reduces mucociliary clearance, and improves drug absorption [10,11]. Furthermore, gellan gum forms a three-dimensional cross-linked network that increases viscosity and acts as a diffusion barrier, thereby controlling drug release and enabling sustained delivery [11]. Regarding liposome-enriched gellan gum systems, previous studies have demonstrated that incorporating liposomes into gellan gum–based in situ gels can enhance formulation performance. Liposomes protect the encapsulated drug from degradation and serve as drug reservoirs, while the gellan gum matrix further modulates release kinetics. Such hybrid systems have been widely investigated for mucosal delivery (nasal and ocular), showing improved mucoadhesion, prolonged retention time, and enhanced bioavailability compared to conventional formulations [12,13]. The integration of liposomes into mucoadhesive in situ gels represents a promising dual-function approach, where the gel matrix ensures prolonged nasal retention and the embedded nanosystems enhance drug diffusion and absorption. This synergistic strategy is particularly suitable for EPL, given its lipophilic nature and compatibility with lipid-based delivery systems.

Therefore, the aim of this study was to develop and optimize an intranasal delivery system for EPL by combining liposomal nanosystems with a mucoadhesive in situ gel matrix. Eplerenone was selected for intranasal delivery to achieve a rapid onset of action by bypassing first-pass hepatic metabolism and enabling direct systemic absorption via the highly vascular nasal mucosa. This approach may also improve bioavailability and potentially allow dose reduction compared to oral administration, which is associated with extensive first-pass metabolism and variable systemic exposure [14,15]. Eplerenone-loaded liposomes (Elip) were first optimized based on critical quality attributes, including mean particle size (MPS), polydispersity index (PDI), zeta potential (ZP), and encapsulation efficiency (EE). The optimized liposomal formulation was subsequently incorporated into a gellan gum–based in situ gel (Elip-GG). The developed formulation was comprehensively evaluated for physicochemical properties (viscosity, pH), mechanical and rheological behavior, in vitro drug release, and safety. Furthermore, in silico computational analysis was performed to investigate molecular interactions and potential biological pathways associated with the developed system.

## 2. Materials and Methods

### 2.1. Excipients and Api

Eplerenone (EPL) was generously supplied by Neutec Pharmaceutical Company, Sakarya, Turkey. Chloroform was sourced from Avantor Performance Materials, Radnor, PA, USA. Sigma Aldrich in Munich, Germany provided sodium chloride, potassium chloride, and calcium chloride, while cholesterol was obtained from Sigma Aldrich Chemie GmbH, Taufkirchen, Germany. Phytagel^®^ (gellan gum) and soybean phosphatidylcholine (SPC) were also procured from Sigma Aldrich, Munich, Germany. Purified water, used for HPLC and sample preparation, was produced with a Millipore Super Purity Water System (Millipore, Burlington, MA, USA). Krebs buffer was acquired from Caisson Lab, Smithfield, UT, USA.

### 2.2. Design-Expert

A D-optimal design was utilized to assess the effects of three independent variables—SPC (A), cholesterol (B), and chloroform (C)—on three response variables: MPS, ZP, and PDI. The parameter ranges used in this study were determined based on research [16]. The software Design-Expert^®^ (version 13, Stat-Ease Inc., Minneapolis, MN, USA) was employed to select the most appropriate model and refine the process. Table 1 outlines the variables and their respective levels used in the design. Analysis of variance tables were produced, and the effects and regression coefficients of various linear models, as well as the correlations between the variables, were calculated. The experimental design resulted in 16 combinations. By comparing several statistical metrics, such as the coefficient of variation, the multiple correlation coefficient (R^2^), and the adjusted multiple correlation coefficient (adjusted R^2^), the optimal mathematical model was identified. The significance of all terms in the polynomial was evaluated by computing the F value at a probability level of *p* < 0.05. Numerical optimization was conducted to refine the fitted polynomials, and the ideal conditions were validated through experiments performed according to the parameters outlined in Table 1.

### 2.3. Preparation of Liposomes

Unloaded liposomes (Ulip): Unloaded liposomes (Ulip) were prepared using the thin-film hydration method as previously described in the literature [17]. SPC and cholesterol were dissolved in chloroform in a round-bottom flask, and the organic solvent was evaporated using a rotary evaporator to form a thin lipid film. The film was hydrated with distilled water and vortexed/sonicated in a bath sonicator for 3 min to facilitate dispersion. The resulting suspension was further processed using probe sonication for 8 min at 70% amplitude with a cycle of 7 s ON and 3 s OFF (7 cycles) to reduce vesicle size. The formulation was then allowed to equilibrate at room temperature (23 ± 0.5 °C) and stored at 4 ± 0.5 °C.

Eplerenone-loaded liposomes (Elip): The Eplerenone-loaded liposomes were also prepared using the thin-film hydration method. SPC, cholesterol, and 12.5 mg of eplerenone were dissolved in a required amount of chloroform, and the same protocol was used as in the preparation of Ulip liposomes.

### 2.4. Characterization Studies of Liposome Formulation

#### 2.4.1. Particle Size Distribution and Zeta Potential

Differential light scattering (DLS) (Nano ZS 3600, Malvern Panalytical Inc., Westborough, MA, USA) was employed to determine the MPS and PDI. Zeta potential (ZP) measurements were conducted using the DLS in zeta mode, with electrocuvettes used for the ZP assessments. The analysis was performed at a temperature of 25 ± 0.5 °C. The samples were diluted with purified water in a ratio of 1:20 [18]. The analysis was done in triplicate, and the obtained results were expressed as a mean ± SD of 10 measurements.

#### 2.4.2. Determination of Encapsulation Efficiency

The encapsulation efficiency (EE) of the liposomes was determined by centrifuging the formulation at 10,000 rpm for 20 min using a laboratory centrifuge (Labofuge 200, Heraeus, Hanau, Germany) to separate the non-encapsulated drug. This indirect method assumes that the free drug remains in the supernatant after centrifugation. The supernatant was then analyzed using high-performance liquid chromatography (HPLC). The EE was calculated using the following formula [19]:

EE (%) = qt − qu/qt × 100

where qu is the amount of unloaded EPL (mg/g) and qt is the total EPL quantity taken (mg/g).

#### 2.4.3. Transmission Electron Microscopy (TEM) Analysis

Before TEM analysis, the loaded and unloaded liposome sample suspension was sonicated for 2 min, and TEM samples were prepared by depositing a drop of the particle suspension on a Formvar/Carbon supported copper grid, staining with 2% uranyl acetate solution for 2 min, and air-drying at room temperature. Stained liposomes were visualized using a transmission electron microscope (TEM, JEOL JEM-2100PLUS, Tokyo, Japan) operated at an acceleration voltage of 200 kV.

### 2.5. Preparation of In Situ Gels

#### 2.5.1. Preparation of Gellan Gum

Gellan gum (GG) solutions were prepared in four different concentrations (0.5%, 0.6%, 0.7%, and 0.8% *w*/*v*) to identify the optimal formulation. The required amount of GG powder was dissolved in 50 mL of purified water in a beaker, stirred continuously with a magnetic stirrer, and set at 500 rpm for 60 min. The preparation was performed at a temperature of 25 ± 0.5 °C.

#### 2.5.2. Preparation of Simulated Nasal Fluid

Simulated nasal fluid (SNF) was prepared by dissolving 0.59 mg/mL of CaCl_2_, 2.98 mg/mL of KCl, and 8.77 mg/mL of NaCl in 100 mL of water. This solution was also stirred with a magnetic stirrer at 500 rpm until clear.

#### 2.5.3. Determination of Gelling Capacity

To determine the optimal concentration of GG, two drops of SNF were added onto glass plates containing different GG formulations. The gelling capacity was evaluated by observing gel formation and its persistence over time.

#### 2.5.4. The Composition and Preparation of In Situ Gels

Liposomal and non-liposomal formulations of EPL-loaded in situ gels were prepared according to the specified ratios outlined in Table 2. For the EPL-GG gel formulations, the gelling agent was initially prepared, followed by dissolving a specified amount of EPL (0.6%) in the appropriate quantity of ethanol, which was then added to the formulation. For the preparation of EPL-loaded liposomal in situ gel formulations, liposomes were initially formulated using EPL, methanol, chloroform, soy phosphatidylcholine (SPC), and cholesterol in defined proportions via the thin-film hydration technique. Following this, the in situ gel base was prepared using gellan gum (GG). The previously prepared liposomes were then incorporated into the gel matrix by mixing at 500 rpm for 30 min at room temperature (25 ± 0.5 °C), employing the direct incorporation method.

### 2.6. Characterization Studies of In Situ Gels

#### 2.6.1. Mechanical Properties

Texture analysis was conducted using a TA-XT Plus Texture Analyser (Stable Micro Systems, Godalming, UK) equipped with a 2 kg load cell in texture profile analysis mode, with a measurement range of 0–100 N. A cylindrical probe with a 15 mm diameter was used as the sample holder. Formulations were placed initially in a Petri dish (3 cm diameter) at room temperature. Each formulation underwent two compressions with an analytical probe to a depth of 50 mm and at a rate of 1 mm/s, with a 15 s delay between the end of the first compression and the beginning of the second. Mechanical parameters such as hardness, compressibility, adhesiveness, and cohesiveness were derived from the resulting force–time curve [20,21,22,23].

#### 2.6.2. Determination of pH of In Situ Gel

A calibrated pH meter (Mettler Toledo, Greifensee, Switzerland) was utilized for measuring the pH of the in situ gel formulations.

#### 2.6.3. Viscosity and Gelling Time

A digital viscometer named Brookfield, equipped with spindle RV72, was used to determine the viscosity of the developed formulations. The formulation’s viscosity was observed at 34 ± 0.5 °C, at 20, 30, and 40 rpm shear rates. The gelling time of the prepared formulation was determined by placing a drop of the formulation in a beaker at 34 ± 0.5 °C, and the gelation time was observed [24].

### 2.7. Fourier Transform Infrared Spectroscopy (FTIR-ATR) Analysis

Attenuated total reflectance (ATR) spectroscopy was used to conduct compatibility studies on the ingredients used in the formulations. Spectra of the formulations were obtained in the wavenumber range of 650–4000 cm^−1^. The formulations were applied directly onto the crystal of the equipment. Multiple scans were performed for each sample, adjusting the force applied to achieve optimal transmittance results [22].

### 2.8. High-Performance Liquid Chromatography Analysis of Eplerenone

HPLC analysis utilized an Agilent 1260 Infinity HPLC system (Agilent Technologies, Santa Clara, CA, USA), comprising a quaternary pump, autosampler, thermostatic column compartment, and diode array detector units. The mobile phase consisted of water, acetonitrile and methanol (65:20:15, *v*/*v*). Ultrapure water for the aqueous mobile phase was generated by a Type I water purification system, specifically the Millipore Simplicity (Darmstadt, Germany). The mobile phase flow rate was maintained at 1.0 mL/min. Separation occurred on an Agilent Poroshell 120 EC-C18 column (2.7 μm, 4.6 × 50 mm) (Agilent Technologies, Waldbronn, Germany) maintained at 40 °C. The injection volume was 10 μL, and EPL was detected at a wavelength of 245 nm.

#### Method Validation

The HPLC method was validated following the ICH guidelines. Validation parameters included specificity, linearity, detection range, accuracy, recovery, precision, and determination of limits of detection (LOD) and quantification (LOQ).

### 2.9. In Vitro Drug Release

EPL release from the formulations (EPL-GG and Elip-GG) was assessed using a Franz diffusion cell (Permegear, Hellertown, PA, USA) with a diffusion area of 3.8 cm^2^ and a receptor volume of 20 mL. A cellulose acetate membrane (Spectra/Por Regenerated Cellulose, MWCO 8–10 kDa) served as the artificial membrane. The receiving compartment contained Krebs buffer solution (pH 6.6) and was maintained at 37 ± 0.5 °C. After adding 2 g of EPL-GG and Elip-GG formulations with 2 mL of Intranasal Simulated Fluid (ISF), the Franz cell’s top was sealed with parafilm. Samples (0.7 mL) were withdrawn every 30 min over 6 h, ensuring no air bubbles entered the receiver compartment, and replaced with an equal volume of fresh medium. HPLC analysis was employed to quantify the drug amounts in the samples.

## 3. Results and Discussion

### 3.1. HPLC Method Validation Studies

#### 3.1.1. Specificity

**Figure 1:**
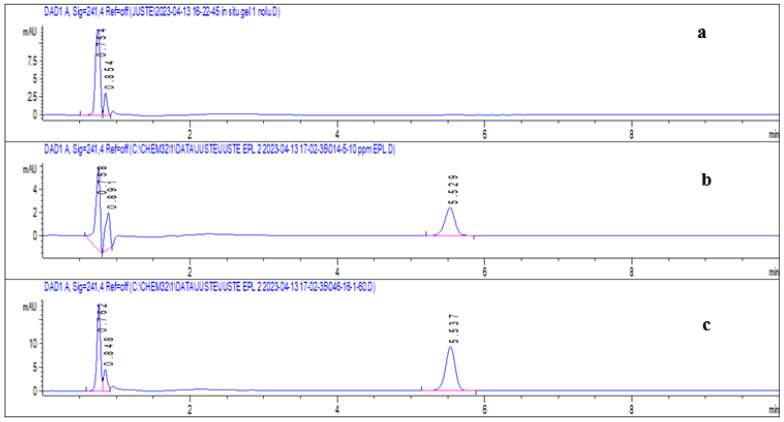
Obtained chromatograms for Eplerenone: (**a**) liposome formulation without Eplerenone; (**b**) liposome formulation with Eplerenone; (**c**) 10 ppm Eplerenone standard solution.

The specificity of the method was evaluated by applying forced degradation studies. For this purpose, EPL solutions were kept under stressed conditions. Different EPL solutions were prepared in 1 M NaOH, 1 M HCl, and 10% hydrogen peroxide. After that, these solutions were boiled in a hot water bath. Also, EPL solutions were kept under UV light. Following these applications, solutions were cooled, filtered, and injected into the HPLC system. It was seen that there was no interference for the EPL peak. Besides this, the specificity of the method was evaluated by comparing a placebo liposome formulation, a liposome formulation containing EPL, and the EPL standard solution (Figure 1). After investigating the chromatograms, it was seen that the other components in the formulation did not show any interference. The peak belonging to EPL was clearly observed.

#### 3.1.2. System Suitability

A system suitability test was applied to understand the suitability of the method. For this purpose, a solution of EPL at a concentration of 15 ppm was injected six times, and capacity factor, resolution, number of theoretical plates, tailing factor, and relative standard deviation of the peak area values were evaluated.

#### 3.1.3. Linearity

The linearity of the method was evaluated over a 1 to 70 ppm concentration range (1, 2, 5, 10, 20, 40, and 70 ppm). Freshly prepared solutions were analyzed in triplicate. The peak areas versus concentration values were used to construct the calibration curve. The correlation coefficient of the method was checked to understand the linearity of the method. Table S2 shows the calibration curve parameters for the method.

#### 3.1.4. Limit of Detection (LOD) and Quantification (LOQ)

An analytical method was established and subsequently validated for the quantification of ELP. The HPLC procedure was assessed in accordance with the ICH Q2 (R1) guidelines [23]. The method demonstrated high sensitivity, with the limit of detection (LOD) and limit of quantification (LOQ) found to be 0.089 µg/mL and 0.29 µg/mL, respectively. These results confirm that the method is suitable and dependable for both the detection and measurement of EPL in pharmaceutical formulations, aligning with ICH regulatory standards.

#### 3.1.5. Accuracy and Precision

The accuracy of the proposed method was evaluated by determining the recovery rates of quality control (QC) samples. This was carried out by analyzing QC samples at concentrations of 3, 15, and 50 ppm, each tested in three replicate sets. To assess precision, the relative standard deviation (RSD) of the recovery values was calculated for both intraday (*n* = 3) and interday (*n* = 9) measurements.

### 3.2. Preparation and Characterization of Unloaded Liposome

A D-optimal experimental design (version 13, Design-Expert^®^ software) was employed to optimize unloaded liposome formulations by evaluating the effects of three independent variables—SPC (A), cholesterol (B), and chloroform (C)—on three critical quality attributes: mean particle size (MPS), polydispersity index (PDI), and zeta potential (ZP), as summarized in Table 3. Liposomes were successfully prepared using the thin-film hydration method and subsequently characterized.

**Figure 2:**
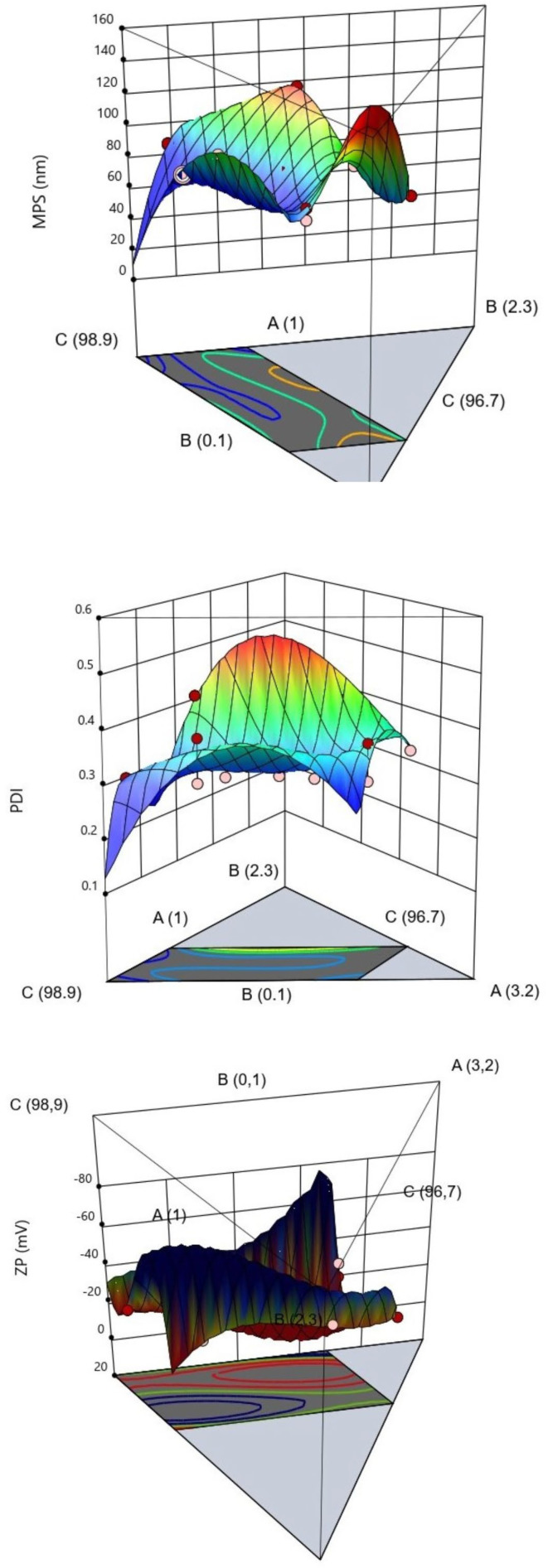
Response surfaces for the effects of liposome ingredients: concentration of SPC (A), cholesterol (B), and chloroform (C) on three critical quality attributes: mean particle size (MPS), polydispersity index (PDI), and zeta potential (ZP).

The experimental matrix consisted of 16 formulation runs, and the corresponding statistical modeling results, including fitted polynomial equations and ANOVA outputs, are presented in Table 4. All model terms were statistically significant (*p* < 0.001), confirming the robustness of the response surface methodology (RSM) approach. The generated models were further visualized using three-dimensional response surface plots (Figure 2), enabling evaluation of factor interactions and their combined influence on the responses.

As shown in Figure 2, the response surface for MPS exhibited pronounced curvature with elliptical contour patterns, indicating significant interaction effects between formulation variables. A distinct minimum region was observed, corresponding to an optimal combination of SPC, cholesterol, and chloroform for minimizing particle size. The presence of steep gradients further suggests that small variations in composition can lead to considerable changes in vesicle size, confirming the nonlinear and multifactorial nature of the system.

The MPS values ranged from 86.76 to 134.43 nm (Table 3), confirming successful formation of nanoscale vesicles suitable for nasal drug delivery. All formulations remained below 135 nm, which is advantageous for enhancing mucosal permeation and improving colloidal stability. Polydispersity index (PDI) values ranged from 0.290 to 0.519, indicating monomodal to heteromodal size distributions throughout the formulations [25,26].

In Figure 2, the PDI response surface demonstrated a relatively smooth and dome-shaped profile, suggesting a predominantly quadratic relationship with less pronounced interaction effects compared to MPS. The contour plots revealed a relatively broad low-PDI region, indicating a stable formulation domain where variations in factor levels do not significantly compromise size distribution uniformity.

All formulations exhibited negative zeta potential values ranging from −21.86 to −42.56 mV (Table 3), suggesting good electrostatic stability and reduced risk of aggregation. The negative surface charge originates from the ionization of phosphatidylcholine headgroups and the adsorption of ionic species at the vesicle surface. Importantly, such a negative charge may also facilitate nasal mucus penetration by minimizing strong electrostatic interactions with negatively charged mucin glycoproteins, which contain sialic acid residues [27,28].

As illustrated in Figure 2, the ZP response surface displayed irregular and steep variations across the design space, indicating a highly sensitive dependence on formulation variables. The non-uniform topology and compressed contour regions suggest strong nonlinear interactions and potential complexity in the underlying mechanisms governing surface charge.

The fitted cubic and quartic polynomial models (ZP, MPS, and PDI) demonstrated strong predictive capability, as shown by high R^2^ and adjusted R^2^ values in Table 4, indicating good model fitness. The presence of significant interaction terms (AB, AC, BC, and higher-order terms) confirms that the formulation variables do not act independently but rather exhibit synergistic and antagonistic effects on liposome characteristics.

In particular, the interaction between SPC and cholesterol (AB) plays a critical role in defining vesicle structure. Cholesterol is known to insert between phospholipid acyl chains, increasing membrane packing density and reducing bilayer fluidity, which enhances vesicle stability while influencing particle size distribution. At optimal concentrations, this interaction stabilizes the bilayer; however, excessive cholesterol may increase rigidity and reduce vesicle flexibility, thereby affecting size and PDI.

Similarly, chloroform (C), as the organic phase solvent, influences lipid dissolution and thin-film formation during liposome preparation. Its interaction with SPC and cholesterol affects the uniformity of the lipid film, thereby impacting vesicle assembly kinetics, size distribution, and homogeneity. These combined effects explain the nonlinear behavior observed in the response surface models.

Final Equations
ZP (Cubic Model)

ZP = 4.89A + 66.91B − 6.77C − 307.34AB − 122.62AC − 271.67BC + 696.21ABC(1)

**MPS (nm) (Quartic Model)**

MPS = −17.90A − 1683.38B + 9.56C + 2592.64AB + 463.98AC + 2683.78BC − 9018.81A^2^BC + 19181.20AB^2^C − 5960.51ABC^2^ + 2949.92AB(A − B)^2^ (2)

PDI (Cubic Model)

PDI = −0.2084A + 9.38B + 0.1288C − 13.79AB + 1.56AC − 14.01BC + 11.03ABC + 16.60AB(A − B)^2^ + 0.2150AC(A − C)^2^ + 8.78BC(B − C)^2^ (3)

The fitted cubic and quartic polynomial models (ZP, MPS, and PDI) demonstrated strong predictive capability, as shown by high R^2^ and adjusted R^2^ values in Table 4, indicating good model fitness. The presence of significant interaction terms (AB, AC, BC, and higher-order terms) confirms that the formulation variables do not act independently but rather exhibit synergistic and antagonistic effects on liposome characteristics. In particular, the interaction between SPC and cholesterol (AB) plays a critical role in defining vesicle structure. Cholesterol is known to insert between phospholipid acyl chains, increasing membrane packing density and reducing bilayer fluidity, which enhances vesicle stability while influencing particle size distribution [29]. At optimal concentrations, this interaction stabilizes the bilayer; however, excessive cholesterol may increase rigidity and reduce vesicle flexibility, thereby affecting size and PDI [30]. Similarly, chloroform (C), as the organic phase solvent, influences lipid dissolution and thin-film formation during liposome preparation. Its interaction with SPC and cholesterol affects the uniformity of the lipid film, thereby impacting vesicle assembly kinetics, size distribution, and homogeneity [31,32]. These combined effects explain the nonlinear behavior observed in the response surface models.

The polynomial equations (Equations (1)–(3)) provide quantitative information on how formulation variables influence liposome properties. Linear terms (A, B, C) describe individual effects, while interaction terms (AB, AC, BC) reflect nonlinear synergistic or antagonistic relationships between SPC, cholesterol, and chloroform. In particular, the negative AB interaction suggests that SPC and cholesterol do not act independently, as cholesterol modifies SPC packing and bilayer rigidity, resulting in nonlinear changes in vesicle size and stability. Similarly, AC and BC interactions indicate that chloroform affects lipid solubilization and thin-film formation, thereby influencing vesicle assembly and size distribution. Higher-order terms in the MPS model (e.g., A^2^BC, AB^2^C, ABC^2^) confirm complex multifactorial behavior during solvent evaporation and hydration, where lipid reorganization is not linear but composition-dependent. Overall, the model demonstrates that liposome characteristics are governed by the combined effects of lipid composition and processing conditions, and the good agreement between predicted and experimental values (*p* > 0.05) confirms the reliability of the optimization model.

Numerical optimization using the desirability function (Table 5) identified the optimal formulation containing 1.78% SPC, 0.42% cholesterol, and 97.8% chloroform. The predicted and experimental values showed excellent agreement with no statistically significant differences (*p* > 0.05), confirming the reliability and predictive accuracy of the developed models (Table 5).

The optimized formulation (Ulip) was subsequently used for drug loading. The resulting EPL-loaded liposomes (Elip) exhibited a mean particle size of 86.56 nm, PDI of 0.29, and zeta potential of −25.43 mV, indicating that nanoscale characteristics were preserved after drug incorporation. The entrapment efficiency of 86.3 ± 3.4% further confirms efficient incorporation of EPL within the liposomal bilayer, suggesting good compatibility between the drug and phospholipid matrix.

Overall, the results demonstrate that the D-optimal design effectively optimized formulation variables and successfully established the relationship between composition and critical quality attributes. The optimized liposomes exhibited desirable nanoscale size, acceptable homogeneity, and sufficient surface charge, making them suitable carriers for subsequent incorporation into the in situ nasal gel system.

### 3.3. Liposomal Morphology

**Figure 3:**
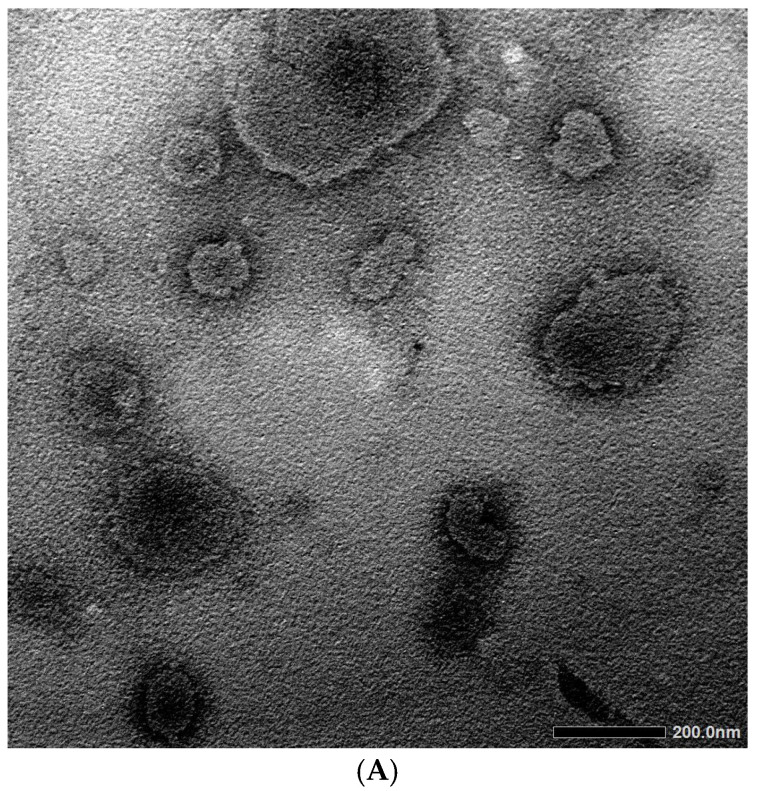

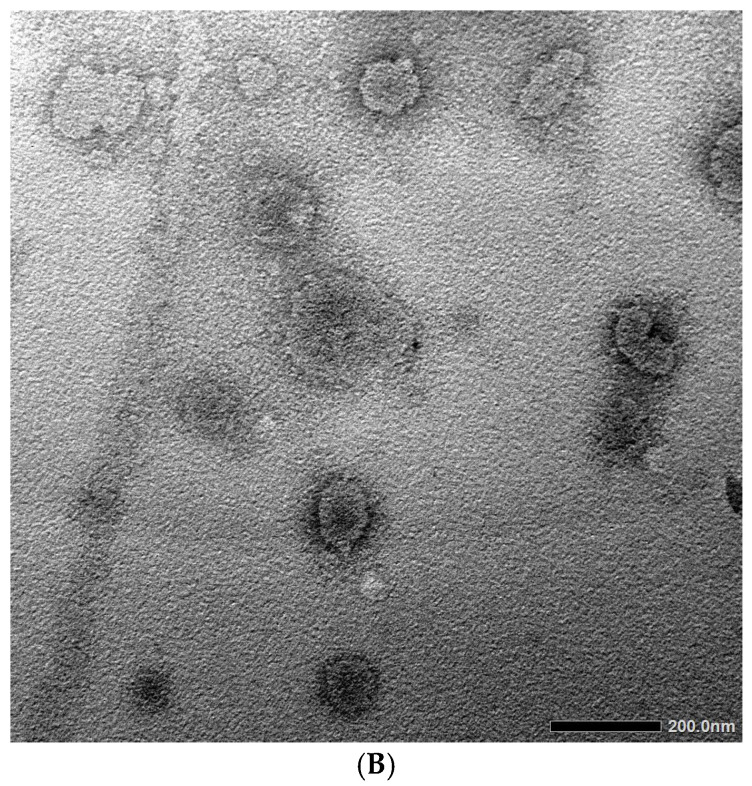
TEM images of optimal Ulip (**A**) and Elip (**B**) formulations. The Ulip formulation contained: 1.78% SPC, 0.42% cholesterol, and 97.8% chloroform; the Elip formulation contained: 1.78% SPC, 0.42% cholesterol, 97.8% chloroform, and 0.6% of EPL.

Transmission Electron Microscopy (TEM) emerges as the most convenient visual technique for investigating the mean particle size and surface morphology of prepared nanoformulations. The surface morphology of the optimal liposomal formulation was assessed through TEM analysis. A TEM image was captured to glean additional insights into the morphology of the prepared formulations (Figure 3).

As depicted in Figure 3, the particles appeared nearly spherical and uniformly sized, with smooth surfaces. These TEM images corroborate the nanosized measurement results, providing supportive data.

### 3.4. In Situ Gel Preparation

The determination of GG concentration was mainly dependent on the viscosity of the gel formed by mixing different concentrations of GG (0.5, 0.6, 0.7, and 0.8%) with an SNF solution. The pH of the in situ gel formulations was 6.84, which was close to the normal physiological pH of the nasal fluid [18]. Formulations containing up to 0.5% *w*/*w* of gellan gum (GG) showed no observable sol–gel transition when exposed to simulated nasal fluid (SNF). This lack of gelation is likely due to the low GG concentration, which may have been insufficient to trigger effective ionic interactions with the SNF. As a result, these samples were not considered for further investigation. In contrast, formulations with 0.6% and 0.7% *w*/*w* GG underwent rapid gel formation upon contact with SNF. This behavior is attributed to the aggregation of double-helical structures facilitated by ionic crosslinking with cations [33]. Conversely, formulations containing 0.8% *w*/*w* GG were deemed impractical due to their excessive solidity. Optimal gel appearance was achieved with a GG concentration of 0.7%. These findings are consistent with those reported by other researchers [20].

### 3.5. In Situ Gel Characterization

#### 3.5.1. Mechanical Properties

Nasal in situ gel formulations typically require appropriate mechanical properties to ensure easy administration and effective mucosal spread [23]. Texture profile analysis (TPA) was conducted to gain insights into the gel structure and evaluate the formulations’ ability to withstand compressive loads and subsequent relaxation. Parameters such as hardness and compressibility were utilized to characterize the mechanical attributes of the formulations (refer to Table 6).

Hardness and compressibility are indicators of the force required to dispense a formulation from its container and describe how the sample behaves under compressive stress. Ideally, these values should be low to ensure effortless removal of the gel [34]. Creating effective intranasal formulations involves optimizing characteristics such as viscosity, mucoadhesion, and spreadability to enhance usability and promote better patient adherence. Texture profile analysis (TPA) is a useful technique for evaluating the mechanical behavior of semi-solid systems by examining their structural integrity [22]. Mechanical parameters, including hardness and compressibility, were obtained from the force–time profiles generated by TPA, with the results summarized in Table 6.

In terms of textural profile analysis, the higher adhesiveness observed in the liposomal in situ gel compared to Ulip-GG and Elip-GG in situ gels could be attributed to increased viscosity. This phenomenon may be explained by the disruption of hydrogen bonds between hydrophilic polymer chains and the solvent, leading to an increase in hydrophobic polymer chains, facilitating micellar aggregation and subsequent polymer gelation [35].

#### 3.5.2. Viscosity

For in situ gelling nasal formulations, achieving an optimal viscosity is essential to ensure both ease of administration and adequate residence time at the application site. The developed formulations are designed to be administered as low-viscosity liquids that undergo rapid gelation upon exposure to the nasal ionic environment through ion-triggered crosslinking of gellan gum [25].

The apparent viscosity of the prepared formulations ranged from 250 ± 0.01 to 3000 ± 0.02 cP depending on formulation composition and applied shear rate (Table 7). A clear decrease in viscosity with increasing shear rate (20–40 rpm) was observed for all samples, confirming non-Newtonian, shear-thinning (pseudoplastic) behavior. This rheological profile is highly desirable for nasal drug delivery systems, as it allows the formulation to flow easily under the mechanical stress generated during spraying while maintaining higher viscosity under low-shear conditions after administration [35].

Such pseudoplastic behavior facilitates efficient atomization during application and enhances spreadability over the nasal mucosa, while simultaneously preventing rapid drainage from the target site. Once deposited, the formulation rapidly recovers its structured state, contributing to prolonged residence time and improved drug retention [35].

Among all tested formulations, liposome-loaded gellan gum systems (Elip-GG and Elip-GG-ANF1) exhibited comparatively higher viscosities. This increase can be attributed to potential interactions between gellan gum chains and liposomal phospholipid components, including hydrogen bonding and physical entanglement within the polymer network, which enhance the overall structural integrity of the system [36].

Regarding gelling performance, formulations exposed to simulated nasal ionic conditions demonstrated marked differences in gelation behavior. Formulations containing an ionic trigger exhibited immediate and stable gel formation (++), whereas formulations without ionic exposure showed no gel formation (−). The rapid gelation observed in the (++) group is characteristic of ion-sensitive gellan gum, which undergoes conformational transition and helix aggregation in the presence of cations, resulting in a firm gel matrix [20].

Overall, the combined rheological and gelling results confirm that all developed formulations possess suitable properties for nasal delivery. The balance between shear-thinning behavior for sprayability and rapid ion-triggered gelation for retention indicates that these systems are well-suited for in situ nasal drug delivery applications [25,35].

#### 3.5.3. pH Measurements

The pH of the prepared in situ formulation is between 6.5 and 6.9, and this result proves that the application can be made without causing any damage to the nasal mucosa (Table 7).

### 3.6. FTIR Analysis

**Figure 4:**
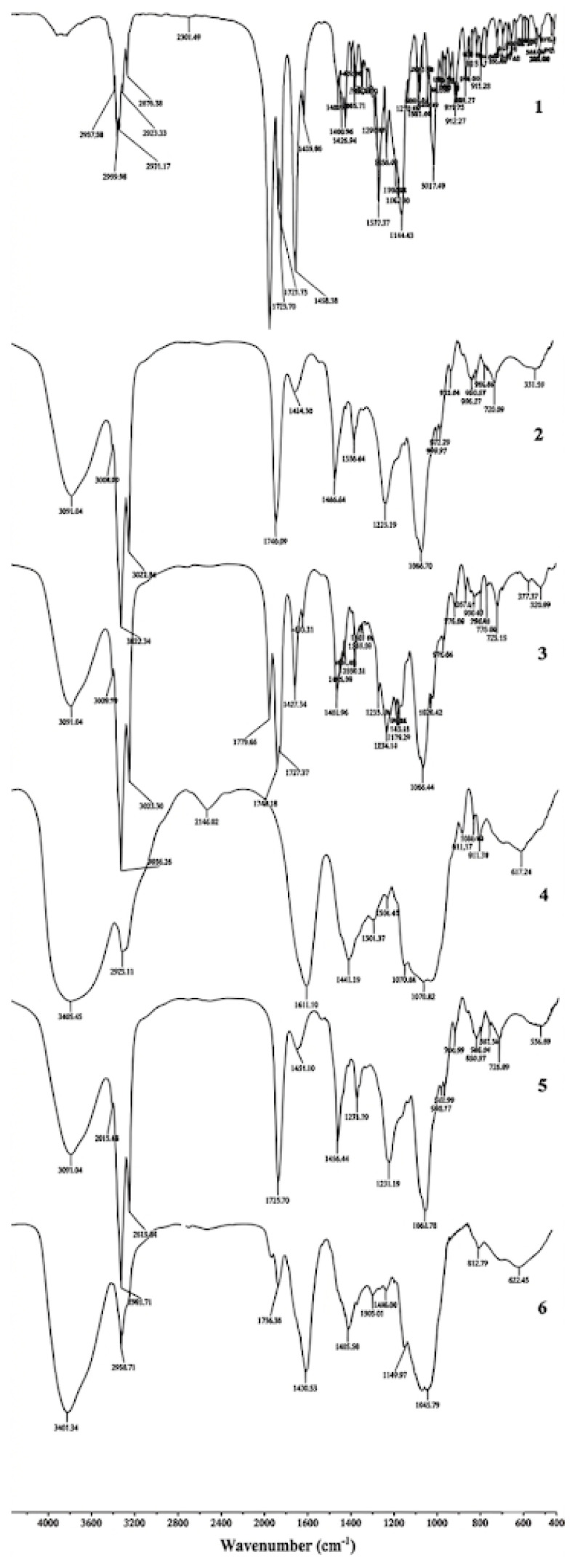
FTIR spectra of active substance and formulations. (1—EPL; 2—Ulip; 3—EPL-loaded liposomes; 4—GG; 5—Ulip-GG; 6—Elip-GG) GG—0.7% of gellan gum; the Ulip-GG formulation contained: 1.78% SPC, 0.42% cholesterol, 97.8% chloroform, and 0.7% of GG; the Elip-GG formulation contained: 1.78% SPC, 0.42% cholesterol, 97.8% chloroform, 0.6% of EPL, and 0.7% of GG; the EPL-GG formulation contained: 0.6% of EPL and 0.7% of GG.

Figure 4 shows the FTIR analysis of EPL and the formulations. The results of the FTIR spectrum of pure EPL in the studies showed that the primary functional group is located at 1725.70 cm^−1^, 1739.72 and <1800 (C=O carbonyl stretching), respectively (Figure 3). These results were found to be consistent with the reference article [37].

The FTIR spectrum of the unloaded liposome is as follows: 3391.12 (NH stretching mode of amine). The major C=O group signal was observed at 1740.67 (Figure 4 and Table 8). The EPL-loaded liposome in FTIR spectra is listed as 1776.85 cm^−1^, 1727.37 cm^−1^, and 1740.18 cm^−1^ (C=O carbonyl stretching). In addition, 3391.91 cm^−1^ corresponds to the NH stretching mode of the amine (Figure 2). The wide range of OH group signals at 3416.78 cm^−1^ is prominent, which belongs to GG (Figure 4 and Table 8). In addition, stretching vibrations belonging to the aromatic C-O group are seen in the spectrum of 2926.42. The signal at 1613.94 cm^−1^ is due to the glycosidic link in GG, which is also prominent (Figure 4 and Table 8). Similarly, a wide range of OH group signals at 3405.45 cm^−1^ is apparent, which belongs to the FTIR spectrum of GG (Figure 2). These results appear to be consistent with the reference study [36].

The FTIR spectrum of Ulip-GG is listed as 3391.04 cm^−1^ (NH stretching mode of amine), and a carbonyl group signal was observed at 1740.96 (Figure 4 and Table 8). Similarly, the FTIR spectrum of Elip-GG is listed as 3431.54 cm^−1^; a wide range of NH group and carbonyl group signals was observed at 1736.28 cm^−1^ [38] (Figure 4 and Table 8).

In the FTIR spectra of Ulip-GG, a wide range of NH signals was observed at 3391.04 cm^−1^. Below 3000 cm^−1^, 2927.49 cm^−1^, 2853.84 cm^−1^ are the sp hydrogens of the structure. Above 3000 cm^−1^, 3009.69 cm^−1^ are the sp2 hydrogens of the structure [38]. Also, a bigger carbonyl group signal was seen at 1740.96 cm^−1^. Similarly, in the FTIR spectrum of Elip-GG, a wide range of NH group signals was observed at 3431.54 cm^−1^. Below 3000 cm^−1^, 2926.71 cm^−1^ are the sp hydrogens of the structure, and when compared with the carbonyl signal of the unloaded liposome, a smaller carbonyl signal was seen at 1736.28 cm^−1^ (Figure 2).

Importantly, the absence of new functional group formation or disappearance of characteristic peaks in the FTIR spectra confirms that no chemical incompatibility or covalent modification occurred during formulation development. This indicates that EPL maintains its chemical integrity within the delivery system, supporting the physical stability of the formulation [39]. From a formulation and drug release perspective, the observed spectral shifts and peak broadening suggest the presence of weak molecular interactions, primarily hydrogen bonding and van der Waals interactions, between gellan gum, phospholipid components, and the encapsulated drug. Such non-covalent interactions are commonly observed in liposomal systems and polymer-based hybrid carriers and are considered beneficial for maintaining structural integrity while enabling controlled drug release [14,40]. These interactions indicate that EPL is not chemically bound to the polymer matrix, which is expected to facilitate controlled but reversible drug release. The drug is primarily entrapped within liposomal vesicles and subsequently immobilized within the three-dimensional gellan gum hydrogel network after in situ gelation. Upon exposure to physiological conditions, drug release is expected to occur through a combination of mechanisms: (i) diffusion of EPL from liposomal vesicles, (ii) gradual relaxation of the hydrogel network, and (iii) penetration of aqueous media into the gel matrix facilitating drug partitioning. Therefore, the FTIR findings collectively support a dual-controlled release mechanism, in which liposomes act as the primary drug reservoir, while the in situ gellan gum gel serves as a secondary diffusion barrier, enhancing residence time and sustaining drug release at the nasal site. This type of hybrid system has been widely reported to improve mucosal retention and prolong drug availability compared to conventional formulations [14].

### 3.7. In Vitro Release Study

**Figure 5:**
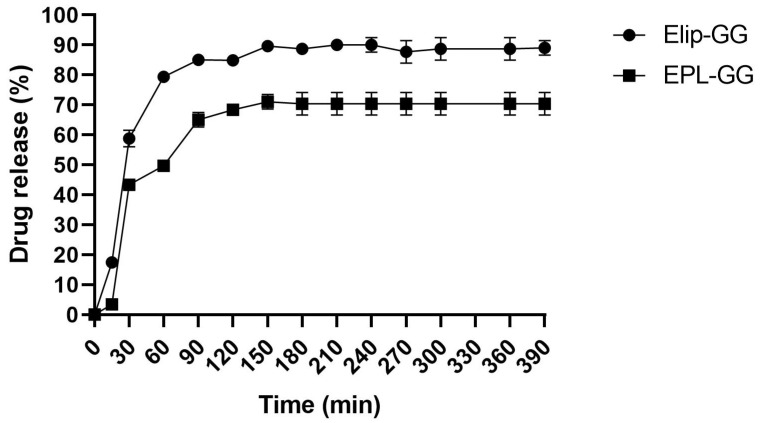
In vitro drug release studies of EPL-loaded formulation, *n* = 3 (the composition of the formulations is shown in Table 1).

The in vitro drug release profiles of EPL-GG (0.6% EPL and 0.6% gellan gum) and Elip-GG (optimized liposomal formulation containing 1.78% SPC, 0.42% cholesterol, 97.8% chloroform, 0.6% EPL, and 0.6% gellan gum) were evaluated using the Franz diffusion cell system. Krebs buffer solution (pH 6.6) was used as the receptor medium to simulate the nasal physiological environment, while a cellulose dialysis membrane served as the diffusion barrier. The cumulative release profiles are presented in Figure 5.

As illustrated in Figure 5, both formulations exhibited an initial rapid release phase followed by a plateau, indicating biphasic release behavior. However, the extent and rate of drug release were markedly different between the formulations. The Elip-GG formulation demonstrated a significantly enhanced release profile, achieving more than 93% cumulative drug release within 180 min, whereas the EPL-GG formulation reached approximately 70% over the same period. This difference was statistically significant (*p* < 0.05), confirming the superior release performance of the liposomal system.

The initial burst release observed in both formulations can be attributed to the diffusion of drug molecules located near or at the surface of the gel matrix. In the case of Elip-GG, this effect is further amplified by the presence of nanosized liposomes, which provide a larger surface area and facilitate faster drug diffusion across the membrane. Following this phase, a sustained release pattern was observed, likely governed by diffusion-controlled mechanisms and gradual drug partitioning from the lipid bilayer into the aqueous medium.

The enhanced drug release from Elip-GG can be primarily attributed to the improved solubilization of EPL within the liposomal structure [41]. The amphiphilic nature of phospholipids and cholesterol contributes to the formation of a bilayer system capable of incorporating both hydrophilic and lipophilic moieties, thereby increasing drug dispersion at the molecular level. Additionally, cholesterol plays a dual role by modulating membrane fluidity and participating in intermolecular interactions, including hydrogen bonding, which can further stabilize the drug within the bilayer while facilitating its controlled release [42].

Furthermore, the nanoscale size of the liposomes (~86 nm) likely contributed to the enhanced release kinetics by reducing diffusion path length and increasing interfacial surface area. The incorporation of liposomes within the gellan gum matrix did not hinder drug release; rather, it appears to have provided a synergistic effect, combining the mucoadhesive and gel-forming properties of the polymer with the solubilization and carrier advantages of liposomes.

Overall, these findings demonstrate that the liposomal in situ gel system (Elip-GG) significantly improves the release characteristics of EPL compared to the conventional gel formulation, highlighting its potential as an effective intranasal drug delivery platform.

Furthermore, a reduced release rate of the active ingredient was observed for the EPL-GG formulation. This behavior can be attributed to the inflection point in the release profile, indicating in situ gel formation within the donor compartment of the diffusion cell. During this process, a fraction of the drug becomes entrapped within the developing gel matrix; consequently, drug release is hindered by the formation of a cross-linked polymeric network and increased viscosity, which collectively limit the diffusion of drug molecules [43].

The in vitro release profile observed in this study is consistent with previously reported characteristics of liposomal and gellan gum–based delivery systems. Liposomes are known to enhance drug release by improving drug solubilization and facilitating diffusion through phospholipid bilayers, often resulting in faster and more complete release compared to conventional formulations [40]. In contrast, gellan gum–based in situ gels undergo ion-triggered gelation, forming a three-dimensional network structure that restricts molecular mobility and reduces drug diffusion rates, thereby providing a sustained release effect as widely reported for nasal and mucosal delivery systems [14].

## 4. Conclusions

This study reports the successful development and optimization of an Eplerenone-loaded liposomal in situ gel (Elip-GG) designed for intranasal administration. The optimized formulation exhibited favorable physicochemical characteristics, including high encapsulation efficiency (86.3%), nanoscale particle size (86.56 nm) with acceptable homogeneity (PDI = 0.29), and a sufficiently negative zeta potential (−29.86 mV), indicating colloidal stability and suitability for mucosal delivery. In vitro release studies demonstrated that the Elip-GG formulation achieved a cumulative drug release exceeding 93% within 2.5 h, which was significantly higher (*p* < 0.05) than that of the control formulation. This enhanced performance can be attributed to the combined effect of liposomal encapsulation, which improves drug solubilization, and the gellan gum–based in situ gel matrix, which modulates drug diffusion and prolongs nasal residence time. Despite these promising findings, it should be noted that the current study is limited to in vitro characterization, and the absence of ex vivo permeation and in vivo pharmacokinetic data restricts definitive conclusions regarding nasal absorption and bioavailability enhancement. Therefore, further investigations are warranted to evaluate mucociliary clearance, nasal permeability, and systemic exposure under physiological conditions. Nevertheless, the Elip-GG system represents a rational and potentially effective strategy for intranasal delivery of Eplerenone and other hydrophilic antihypertensive agents. This platform may offer advantages over conventional oral and parenteral routes by improving drug residence time, enabling controlled release, and potentially enhancing therapeutic efficacy while reducing systemic variability. Future studies should focus on comprehensive in vivo evaluation and long-term stability to support clinical translation.

## Figures and Tables

**Table 1:** Optimal design component variables for the preparation of Eplerenone-loaded liposomes.

Run No.	Independent Variables		
	* A: SPC, (%)	B: Cholesterol, (%)	C: Chloroform, (%)
1	2.5	0.21	97.29
2	1.83	0.51	97.66
3	1.53	0.46	98.01
4	1.55	0.10	98.35
5	1.83	0.51	97.66
6	2.22	0.13	97.65
7	2.10	0.74	97.16
8	1.52	0.80	97.68
9	1.55	0.10	98.35
10	2.50	0.80	96.70
11	2.50	0.21	97.29
12	1.89	0.10	98.01
13	1.00	0.34	98.66
14	1.00	0.34	98.66
15	1.18	0.80	98.02
16	1.83	0.51	97.66

* SPC: soybean phosphatidylcholine.

**Table 2:** The composition of the in situ gel formulations.

Formulation Code	EPL (%)	GG (%)	SPC (%)	Cholesterol (%)
EPL-GG	0.6	0.7	_	_
Elip-GG	0.6	0.7	1.78	0.42
ULip-GG	_	0.7	1.78	0.42

The EPL-GG formulation contained: 0.6% of EPL and 0.7% of GG; the Elip-GG formulation contained: 1.78% SPC, 0.42% cholesterol, 97.8% chloroform, 0.6% of EPL and 0.7% of GG; the Ulip-GG formulation contained: 1.78% SPC, 0.42% cholesterol, 97.8% chloroform, and 0.7% of GG.

**Table 3:** The response variables for the preparation of unloaded liposomes.

Run No	MPS nm	PDI	ZP mV
1	89.81	0.298	−27.63
2	93.21	0.306	−31.81
3	95.40	0.291	−30.30
4	86.76	0.303	−27.53
5	94.00	0.290	−31.81
6	100.60	0.309	−32.80
7	104.66	0.419	−30.96
8	134.43	0.519	−32.60
9	93.16	0.385	−27.10
10	99.36	0.319	−42.56
11	96.90	0.368	−21.86
12	110.00	0.400	−29.23
13	87.00	0.298	−25.53
14	86.31	0.298	−25.53
15	102.63	0.425	−37.16
16	93.21	0.306	−31.81

MPS—mean particle size; PDI—polydispersity index; ZP—zeta potential.

**Table 4:** Fitting models, equations, and statistical parameters of the experimental design.

Response	Model	R^2^	Adjusted R^2^	Predicted R^2^
ZP	Cubic	0.8985	0.8308	0.5433
MPS (nm)	Quartic	0.9516	0.8962	0.8063
PDI	Cubic	0.8099	0.6832	0.4296

**Table 5:** Numerical optimization of material amounts using desirability function.

Independent Variables		
	Amount of Level	Predicted Optimal Amount
SPC (%)	1–2.5	1.78
Cholesterol (%)	0.1–0.8	0.42
Chloroform (%)	96.7–98.9	97.80
**Response Variables**		
**Responses**	**Predicted Mean Value**	**Obtained Mean Value**
MPS (nm)	86.31	97.97
PDI	0.29	0.346
ZP	−29.86	−30.39

SPC—soybean phosphatidylcholine; MPS—mean particle size; PDI—polydispersity index; ZP—zeta potential.

**Table 6:** Mechanical properties of the in situ gel formulations.

Formulation Code	Hardness (g)	Compressibility (g·s)
GG	14.15 ± 0.51 ^b^	32.76 ± 0.24 ^b^
Ulip-GG	10.87 ± 0.32 ^c^	25.85 ± 0.31 ^d^
Elip-GG	10.18 ± 0.42 ^d^	28.06 ± 0.23 ^c^
EPL-GG	10.52 ± 0.23 ^d^	27.38 ± 0.74 ^c^

GG—0.7% of gellan gum; the Ulip-GG formulation contained: 1.78% SPC, 0.42% cholesterol, 97.8% chloroform, and 0.7% of GG; the Elip-GG formulation contained: 1.78% SPC, 0.42% cholesterol, 97.8% chloroform, 0.6% of EPL, and 0.7% of GG; the EPL-GG formulation contained: 0.6% of EPL and 0.7% of GG. Data presented as means ± SD, *n* = 6. Different letters in each column denote statistical differences at *p* ≤ 0.05.

**Table 7:** Viscosity ranges and gelling capacity from different formulations.

Formulation Code	20 rpm (cp)	30 rpm (cp)	40 rpm (cp)	Gelling Capacity	pH Values
GG	1500 ± 0.01 ^d^	667 ± 0.01 ^d^	333 ± 0.01 ^d^	−	6.84 ± 0.23
GG-ANF ^1^	3000 ± 0.02 ^a^	2000 ± 0.02 ^a^	1500 ± 0.02 ^a^	++	−
Ulip-GG	1000 ± 0.03 ^e^	667 ± 0.02 ^d^	250 ± 0.01 ^c^	−	6.63 ± 0.25
Ulip-GG-ANF ^1^	2000 ± 0.04 ^c^	1667 ± 0.04 ^c^	1500 ± 0.02 ^a^	++	−
Elip-GG	1500 ± 0.01 ^d^	1000 ± 0.03 ^b^	500 ± 0.03 ^b^	−	6.54 ± 0.27
Elip-GG-ANF ^1^	2500 ± 0.02 ^b^	2000 ± 0.02 ^a^	1500 ± 0.01 ^a^	++	−
EPL-GG	1500 ± 0.01 ^d^	1000 ± 0.01 ^b^	750 ± 0.03 ^b^	−	6.65 ± 0.22
EPL-GG-ANF ^1^	2700 ± 0.04 ^a^	2000 ± 0.03 ^a^	1500 ± 0.04 ^a^	++	−

(−) no gel formation; (++) immediate and stable gel formation upon exposure to simulated nasal ionic conditions. ^1^ The mixture was half liposome and half artificial nasal fluid (Anf). GG—0.7% of gellan gum; the Ulip-GG formulation contained: 1.78% SPC, 0.42% cholesterol, 97.8% chloroform, and 0.7% of GG; the Elip-GG formulation contained: 1.78% SPC, 0.42% cholesterol, 97.8% chloroform, 0.6% of EPL and 0.7% of GG; the EPL-GG formulation contained: 0.6% of EPL and 0.7% of GG. *n* = 6, ^a–e^ the different letters show a statistical difference between the values, *p* < 0.05.

**Table 8:** FTIR spectral wavenumbers of EPL and formulations.

Sample	Wavenumber (cm^−1^)
Pure EPL	1725.70, 1739.72, <1800
Unloaded Liposome (Ulip)	3391.12, 1740.67
EPL-loaded Liposome (Elip)	1776.85, 1740.18, 1727.37, 3391.91
Gellan Gum (GG)	3416.78, 3405.45, 2926.42, 1613.94
Ulip-GG	3391.04, 1740.96
Elip-GG	3431.54, 1736.28

## Data Availability

Data is contained within the article.

## Supplementary Materials

The following supporting information can be downloaded at: https://www.mdpi.com/article/10.3390/pharmaceutics18060678/s1, Table S1: System suitability parameters for 15 ppm EPL solution; Table S2: Calibration curve parameters for the analysis of EPL; Table S3: Accuracy and Precision of the Method for EPL.

## Abbreviations

The following abbreviations are used in this manuscript:

BCS	Biopharmaceutical Classification System
DLS	Differential light scattering
EE	Encapsulation efficiency
Elip	Eplerenone-loaded liposome
Elip-GG	Eplerenone-loaded liposome incorporated into an in situ gel
EPL	Eplerenone
FTIR	Fourier Transform Infrared Spectroscopy
GG	Gallan gum
HPLC	High-performance liquid chromatography
MPS	Mean particle size
NSS	Nanosystems
PDI	Polydispersity index
SNF	Simulated nasal fluid
SPC	Soybean phosphatidylcholine
TEM	Transmission Electron Microscopy
TPA	Texture profile analysis
ZP	Zeta potential
