# Supplementary material for: Development and Optimization of an Eplerenone-Loaded Liposomal In Situ Gel for Enhanced Intranasal Delivery
Source: Pharmaceutics. 2026 May 29;18(6):678. doi: 10.3390/pharmaceutics18060678 (PMC13305314; doi:10.3390/pharmaceutics18060678)
Supplement: Supplementary file 1 [file pharmaceutics-18-00678-s001.zip › pharmaceutics-4234264-supplementary.pdf]

Supplementary Materials: Development and Optimization of an Eplerenone-Loaded Liposomal In Situ Gel for Enhanced Intranasal Delivery

Juste Baranauskaite, Ipek Ceken, Asta Kubiliene, Rima Jurate Gerbutaviciene, Ebru Türköz Acar and Cetin Tas

Table S1. System suitability parameters for 15 ppm EPL solution.

Parameter	Recommendation	Value
**Capacity Factor (k′)**	k′ > 2	5.877
**Resolution (Rs)**	Rs > 2	17.884
**Theoretical Plates (N)**	N > 2000	2475
**RSD (Peak Area)**	RSD ≤ 2	1.203
**Tailing Factor (T)**	T ≤ 2	0.840

Table S2. Calibration curve parameters for the analysis of EPL.

Parameter	Value
**Equation**	y=18.801x + 2.7741
**R^2**	0,9999
**Concentration Range**	1-70 ppm
**Standard deviation of slope**	0.525834
**Standard deviation of intercept**	0.560728
**LOD**	0.089473
**LOQ**	0.298244

Table S3. Accuracy and Precision of the Method for EPL.

		Intraday Studies (n=3)		Interday Studies (n=9)	
	**Concentration (ppm)**	**Recovery**	**RSD**	**Recovery**	**RSD**
**LLQC**	3	96.2	0.31	96.5	1.19
**MLQC**	15	99.6	0.28	99.3	1.64
**HLCQ**	50	99.9	0.11	99.4	177
